# Advantages of the zebrafish tumor xenograft model: the evaluation of efficacy in cancer therapy and the application to the study of lncRNAs

**DOI:** 10.3389/fimmu.2024.1483192

**Published:** 2024-09-30

**Authors:** Chengwu Hu, Ling Sun, Jianqing Chen, Zhengbing Lyu, Chen Yuan, Xiaofeng Jiang

**Affiliations:** ^1^ College of Life Sciences and Medicine, Zhejiang Sci-Tech University, Zhejiang Provincial Key Laboratory of Silkworm Bioreactor and Biomedicine, Zhejiang Sci-Tech University, Hangzhou, China; ^2^ Pediatric Department, The First People’s Hospital of Huzhou, Huzhou, China

**Keywords:** zebrafish model, anti-tumor research, cancer therapy, lncRNAs, immunotherapy

## Abstract

In the current preclinical anti-tumor researches, there is a general lack of an *in vivo* model that can quickly and efficiently screen effective anti-tumor drugs. As a species that is 87% genetically similar to humans, zebrafish have been widely used to model human diseases, and they are considered an alternative economic model for studying cancer development, proliferation, and metastasis. The zebrafish tumor xenograft model has been effectively used for cancer drug development at all levels, including target validation, and high-throughput screening of long non-coding RNAs (lncRNAs) that may be involved in tumor regulation. In this review, we provide a comprehensive overview of zebrafish as an *in vivo* model for cancer cell growth, migration, anti-tumor immunotherapy, and anti-tumor drug screening. In addition, the regulatory mechanisms of some active lncRNAs have been identified to play a role in the pathogenesis of cancer, but it is still necessary to take advantage of the efficient zebrafish model to screen and learn more about the role of these molecules in tumor development and migration. Current anti-tumor therapies are limited by severe toxicity and multidrug resistance. There is an urgent need for the cost-effective and efficient *in vivo* research tools to improve our understanding and overcome these problems. This paper reviews the different purposes of anti-tumor research using zebrafish model. We discuss the use of zebrafish in cancer cell proliferation and metastasis, identifying signaling pathways, cancer drug discovery and treatment development, and toxicity studies. Finally, this review highlights the limitations of the field and future directions to effectively utilize zebrafish as a highly efficient model for cancer treatment development.

## 1 Introduction

Cancer is a disease with complex causes, including genetic mutation, chromosomal translocation and deletion, amplification, and epigenetic alteration (1). While cancer death rates continue to decline, some cancer types are on the rise, such as breast, pancreatic, and uterine cancers, which are increasing in the United States by 0.6% to 1% per year between 2015 and 2019. The incidence of prostate cancer, liver cancer (in women), kidney cancer, human papillomavirus-associated oral cancer, and melanoma is increasing by 2% to 3% per year. The incidence of cervical cancer (30-44 years old) and colorectal cancer (<55 years old) in young people are also increasing at a rate of 1-2% per year (2). Similarly, by 2022, there will be approximately 4,824,700 new cases of malignant tumors and 2,574,100 deaths in China, and the burden of disease is still rising (3). Thus, it remains a major health problem, and effective animal models and therapeutic studies of many types of cancer are lacking. Noncoding RNAs (ncRNAs) differ from mRNAs in that most of them are not translated into proteins and are generally categorized according to the number of bases, of which those larger than 200 bases are called lncRNAs. LncRNAs are important and powerful cis- or trans-regulators of gene activity, acting as scaffolds for chromatin-modifying complexes and nucleosomes, as well as enhancers and mediators of remote chromatin interactions (4–7). A study analyzing the expression profiles of lncRNAs (and other ncRNAs) in samples from patients with a variety of cancers and comparing them with corresponding normal cells showed that many lncRNAs are dysregulated in various types of cancer (8). In prostate cancer, genome-wide RNA-Seq analysis identified many lncRNAs that are up- or down-regulated in prostate cancer, such as PCA3, PCGEM1, and PCAT-1 are highly associated with prostate cancer (9). In breast cancer, lncRNAs involved include HOTAIR, ANRIL, ZFAS1, NEAT1, DANCR, HIF1A-AS, XIST, TOPORS-AS1, LSINCT-5, and LNP1 (10). A number of deregulated lncRNAs have also been identified in other different types of cancers. Tumor research has focused on lncRNAs because of their functional diversity.

Zebrafish (Danio rerio) has become one of essential vertebrate model organisms for biomedical research. Zebrafish xenograft models offer many unique benefits for cancer research, including the optical transparency of zebrafish embryos and juveniles, which allows for *in vivo* observation and assessment of proliferation and migration at 96 h post-fertilization (hpf) (11). Zebrafish is a reliable model organism for studying tumor growth and metastasis (12, 13), and zebrafish models can be applied to the study of the role of lncRNAs in tumor development. For instance, the roles of lncRNA SNHG4 in the proliferation and migration of colorectal cancer (CRC) (14) and THOR in melanoma (15) have been explored. Zebrafish has emerged as an effective experimental animal model in the transition from *in vitro* experimental models, such as cells, to mammalian models, such as mice, and is valuable to drug-active molecule screening, antitumor drug development, tumor immunotherapy, and research on the functions of tumor-associated lncRNAs (16–18). In this paper, we mainly review and summarize some zebrafish xenograft tumor models and their uses in the study of tumorigenesis, proliferation, migration, and antitumor mechanism based on lncRNAs.

## 2 Model organism - zebrafish

### 2.1 Emergence of zebrafish as a model organism

Zebrafish is a common vertebrate model organism. George Streisinger first recognized the potential of zebrafish as a vertebrate model organism in the early 1980s and published the first paper describing the use of zebrafish as a vertebrate model (19). His research provided a practical basis for the use of zebrafish as a vertebrate model for humans and opened the door to the study of zebrafish. Continuous and in-depth research on zebrafish in the following decades has gradually recognized the potential of zebrafish as a model organism for the study of human diseases. Researchers from the United States and Germany completed the screening of mutated genes in zebrafish in 1996 (20, 21), and the whole genome of zebrafish was sequenced in 2000. Providing insights into the genetic background of zebrafish, several zebrafish models of human diseases have been established. Since then, many zebrafish disease models have been established, demonstrating the importance of zebrafish as a model organism.

### 2.2 Developmental processes and stages of zebrafish

**Figure 1:**
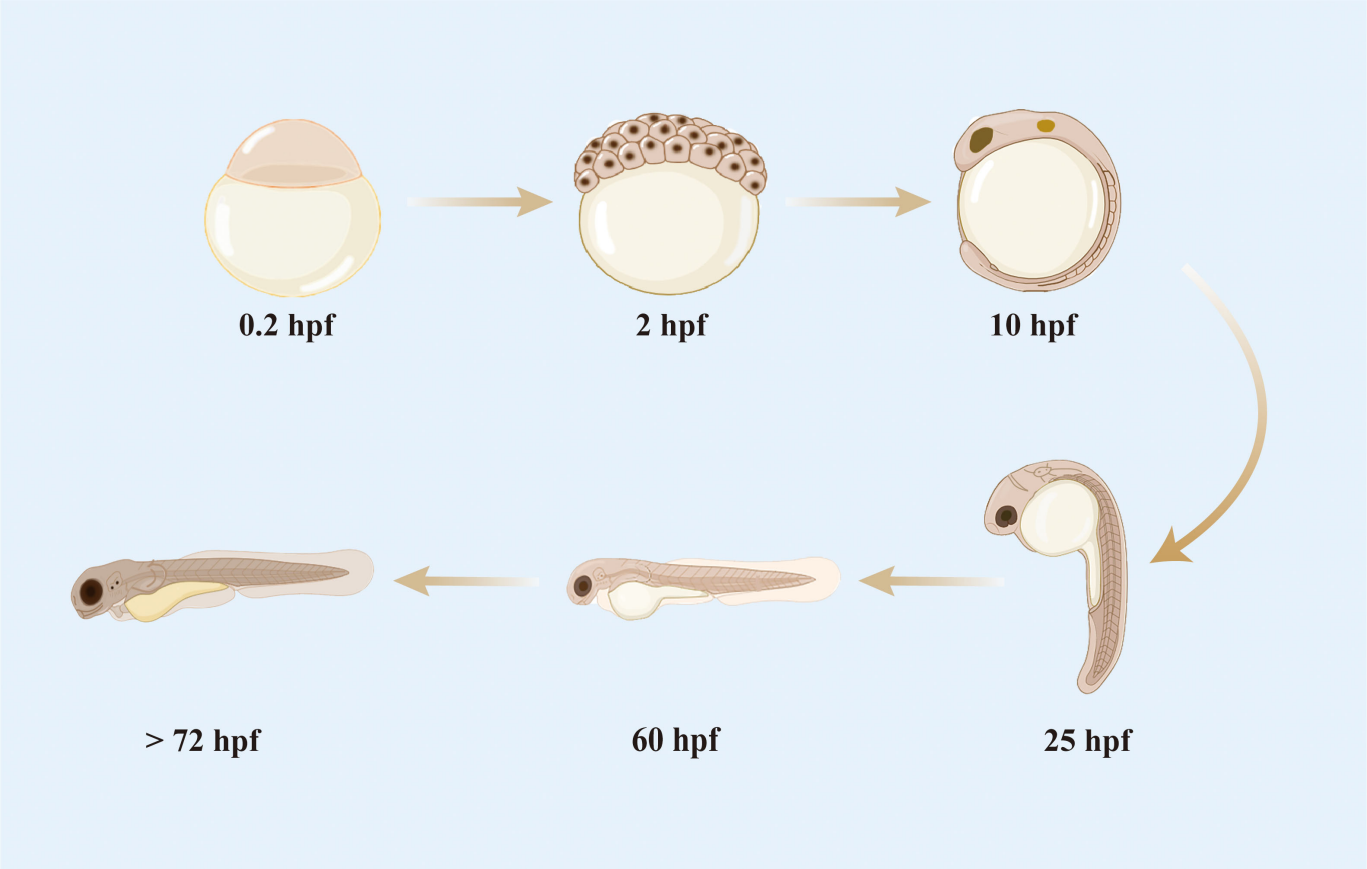
Development process of zebrafish embryo.

Zebrafish has highly similar development process to the embryos of higher vertebrates, including humans (22–24). Compared with mice, zebrafish are small and easy to manipulate for gene editing and reproduction and can be bred in large numbers at a low cost. The embryonic development of zebrafish is rapid (**Figure 1**); a single-celled fertilized egg develops into a motile and transparent embryo within 24 h. The majority of morphological changes are completed at 3 days post-fertilization (dpf) (25), and the digestive system and mouth are functional between 5 and 6 dpf. The yolk sacs throughout the embryonic and early larval stages are rapidly depleted and fully absorbed by 7 dpf. Once a zebrafish develops most of its features, it is considered a juvenile; when it is capable of producing living gametes and reproducing, it is considered an adult (25, 26). Under optimal culture conditions, laboratory zebrafish reach sexual maturity in the third month of their development.

### 2.3 Merits of zebrafish as a model in tumor research

A model organism necessarily requires an extremely high degree of conservation of its target genes with respect to the object of a study. Many factors involved in tumor progression are highly conserved between zebrafish and humans. Approximately 71.4% of human genes have at least one zebrafish homolog, and 82% of human disease-related genes have at least one zebrafish homolog (27). In addition, multiple epigenetic marks regulating gene expression are conserved in vertebrates, including zebrafish and humans (28). Despite having cell cycle genes, zebrafish has conserved tumor suppressors and oncogenes, which facilitate studies on oncogenic pathways and targeted tracing in zebrafish models. Moreover, due to the ease of introducing exogenous genes by microinjection at the single-cell stage of zebrafish, as well as the transparent development of the zebrafish embryo, where the expression and distribution of exogenous genes are easy to visualize and detect (29), zebrafish can be easily genetically manipulated through knockouts, overexpression, and transgenesis, allowing for the creation and characterization of cancer models at the genetic level (30). Notably, a high degree of similarity has been observed between the histopathology of human primary tumors and tumors transplanted in zebrafish (31). However, some genes involved in human cancer progression do not have clear homology in zebrafish, including leukemia inhibitory factor, oncostatin M, and breast cancer susceptibility genes (27).

## 3 Zebrafish tumor xenograft model and its application

### 3.1 Tumor xenograft models in zebrafish

The zygotes in zebrafish can develop rapidly *in vitro* and exhibit a certain degree of transparency. These features allow for the direct visualization, microscopic manipulation throughout embryogenesis and larval development, and observation of fluorescently labeled tumor cell lines with a microscope, thereby facilitating studies on the biology of tumor cells in living organisms. These merits establish zebrafish as effective tumor models. In addition, zebrafish shows early embryonic immune system defects at 0–5 dpf, when the adaptive immune system is still underdeveloped; these defect results in the poor development of the thymus in adult zebrafish, enabling it to tolerate exogenous cell implantation (32). Moreover, no rejection reaction occurs when xenogenic tumor transplantation is carried out in this period. A human tumor xenotransplantation model in fish has many benefits.

In 2005, Lee et al. (33) or the first time implanted human melanoma cells into zebrafish through xenotransplantation, providing a theoretical basis and experimental foundation for the construction of zebrafish xenotransplantation model. In 2007, Stoletov et al. (34), constructed a zebrafish juvenile xenotransplantation model by manipulating a xenotransplantation model on a 1 dpf zebrafish embryo, demonstrating that zebrafish larvae possess a window of observation for tumor formation, cell invasion, and tumor-induced angiogenesis. Tumor transplantation in zebrafish has emerged as an important model for oncology research over the past few years (35), and many important genes and pathways involved in cancer formation are highly conserved between the two species due to the high similarity between zebrafish and humans in terms of genetic background (31, 36). Through the study of zebrafish tumor xenograft models, the molecular mechanisms of human tumorigenesis and development, the formation of tumor microenvironment, and the therapeutic effects of antitumor drugs and therapies have been explored in depth.

The current application of zebrafish to the construction of various benign and malignant tumor models involves direct oncogenic drug intervention, gene mutation, and tumor cell transplantation, among which there is a rich variety of xenotransplantation models, such as leukemia, melanoma, glioma, and hepatocellular carcinoma models (**Table 1**). Xenotransplantation is simpler than chemical, transgenic, and genetic mutation. A specific zebrafish tumor model can be established by injecting tumor cells through xenotransplantation. The body of a zebrafish is transparent before adulthood, and thus tumor growth can be directly observed with a body microscope. An adult zebrafish can be observed under a live imager, and the size and position of fluorescently labeled tumor growth can be observed and recorded in real time. In the preparation of zebrafish xenotransplantation models, a large number of model zebrafish can be obtained simultaneously for parallel evaluation and analysis. Tumor models with site and size differences can be constructed by controlling the number and location of injected tumor cell, and tumor models with special properties by modifying tumors. Moreover, the construction of 2–5 day models do not require immunosuppression, thus simplifying experimental procedures and shortening the experimental period.

**Table 1:** Tumor xenograft models in zebrafish.

Strain	Tumor Type	Time	Cell number	Injection site	Reference
Tg (fli1: eGFP)	Breast cancer	2 dpf	50 - 400	duct of Cuvier	(37)
Tg (fli1: eGFP)	glioblastoma	30 dpf	2×10^5^	cerebrum	(38)
Tg (fli1: eGFP)	glioblastoma	30 dpf	2×10^5^	cerebrum	(38)
Tg (fli1: eGFP)	neuroendocrine tumor	2 dpf	100	perivitelline space	(39)
Tg (fli1: eGFP)	pancreatic cancer	2 dpf	200	yolk	(40)
Tg (fli1: eGFP)	pituitary adenoma	2 dpf	100	perivitelline space	(41)
AB	rhabdomyosarcoma	>30 dpf	2×10^4^	intraperitoneal cavity	(42)
Casper	multiple myeloma	2 dpf	100	perivitelline space	(43)
ABTL, Albino, Casper, Tg (fli1: EGFP), Tg (mpo: GFP)	ewing sarcoma	2 dpf/30 dpf	500 - 800	yolk/eye	(44)
Casper	leukaemia	2 dpf	25 – 50	yolk	(45)
AB, achesb55, mutant	liver cancer	2 dpf	300	yolk	(46)
AB, Casper	pancreatic	2 dpf	50 - 80	yolk	(47)
AB	Adrenocortical cancer	2 dpf	240	yolk	(48)
Tg (fli1: eGFP)	Adenocarcinoma of the lungs	2 dpf	200 - 300	yolk	(49)
Tg (fli1: EGFP)	Oral squamous cell carcinoma	2 dpf	50	yolk	(50)
Tg (Kdrl: mCherry)	liposarcoma	2 dpf	50 - 400	heartbeat	(51)

### 3.2 Applications in tumor angiogenesis and migration

Given that angiogenesis of tumor tissues plays a key role in tumor growth and metastasis (52), observing and studying the angiogenic process of tumor tissues is of great value in studies on tumor production and migration. In 2001, Sumio Isogai et al. (53) found that the blood vessels of zebrafish and other vertebrates have great similarities, showing that studying the process of tumor angiogenesis in zebrafish is effective and feasible and providing an experimental basis for studying human tumor angiogenesis in zebrafish.

Zebrafish has benefits for evaluating the process of tumor angiogenesis, including its early embryonic transparency, high reproductive efficiency, and fast metabolic efficiency. Thus, it is more suitable for research on tumor angiogenesis than rabbits, mice, and chicken embryos and contributes to the rapid developments in this field (54). In addition, the application of transgenic angiofluorescent strain of zebrafish allows for the direct *in vivo* real-time evaluation of angiogenesis-inhibiting drugs on the neovascular system of tumor tissues, facilitating the rapid and precise screening and effect analysis of anti-angiogenic drugs (55). The many features of zebrafish facilitate research on the kinetics of microtumor formation and neovascularization through high-resolution imaging. Meanwhile, the interaction between fluorescent tumor cells and GFP-labeled host vasculature can be described in detail by the three-dimensional reconstruction of confocal microscopy images (56). This model system provides a clear window for observing the mechanisms of microtumor formation, cell invasion, and tumor-induced angiogenesis in mature animals. On the other hand, the process of vascular system generation of transplanted tumors in juvenile fish may be close to the process of tumor angiogenesis in cancer patients (57).

### 3.3 Applications in tumor immunotherapy

Unlike the vascular system that supports circulation, lymphatic vessels form a blind-ended tree-like structure rather than a circulatory loop. Tumors generate not only blood vessels but also lymphatic vessels in the microenvironment. Immune and inflammatory cells in lymphatic vessels enter the tumor microenvironment through infiltration, prompting the body to use autoimmunity against a tumor (58). The molecular mechanisms controlling lymphatic vessel development are largely conserved in zebrafish compared with humans, and lymphatic vessels in both involve key factors such as *vegfc*, *vegfd*, *vegfr3*, *ccbe1*, and *prox1a* during development (59). Therefore, the same characteristics of zebrafish embryonic transparency can be utilized in observing the transgenic zebrafish fluorescently labeled with lymphatic vessels in real time under an imager and studying the status of lymphatic vessel generation in the tumor microenvironment, serving as a platform for the observation of metastasis, proliferation, and dissemination of tumor cells via lymphatic vessels.

Based on the generative mechanism of the tumor microenvironmental lymphatic vascular system in zebrafish xenograft models, this model can be used in exploring tumor immunotherapy and provides insights for clinical tumor therapies, such as immune checkpoint inhibitor drugs. Representative drugs in this class include CTLA-4 inhibitors (60) and PD-1 inhibitors (61). Given that zebrafish have similar immune molecular mechanisms to humans, a portion of immune checkpoint inhibitory drugs can be used in zebrafish for studies on relevant drug mechanism of action and efficacy.

CAR-T therapy is one of the methods for tumor immunotherapy. In this method, T-lymphocytes extracted from a tumor lesion site can be tagged labels that recognize tumor markers, expanded, cultured *in vitro*, and re-infused back to a patient’s body. These cells can specifically kill tumor cells. As zebrafish progressively produce immune-related cells, such as T-cells, B-cells, and natural killer cells, as they develop into larvae, and gradually form a complete innate and acquired immune system, CAR-T therapy can be applied to zebrafish xenograft models for vivo studies. Susana Pascoal et al. (62) demonstrated that CAR-T cell–mediated elimination of target cells can be monitored in zebrafish embryos and CD19-specific CAR-T cells can kill pre-B leukemia cells (Nalm-6) in zebrafish embryos; in addition, they developed a system for quantifying changes in tumor and CAR-T cells over time; they suggest that a zebrafish xenograft model allows for the low-cost and rapid preclinical evaluation of CAR-T cells.

### 3.4 Application in tumor drug screening

**Figure 2:**
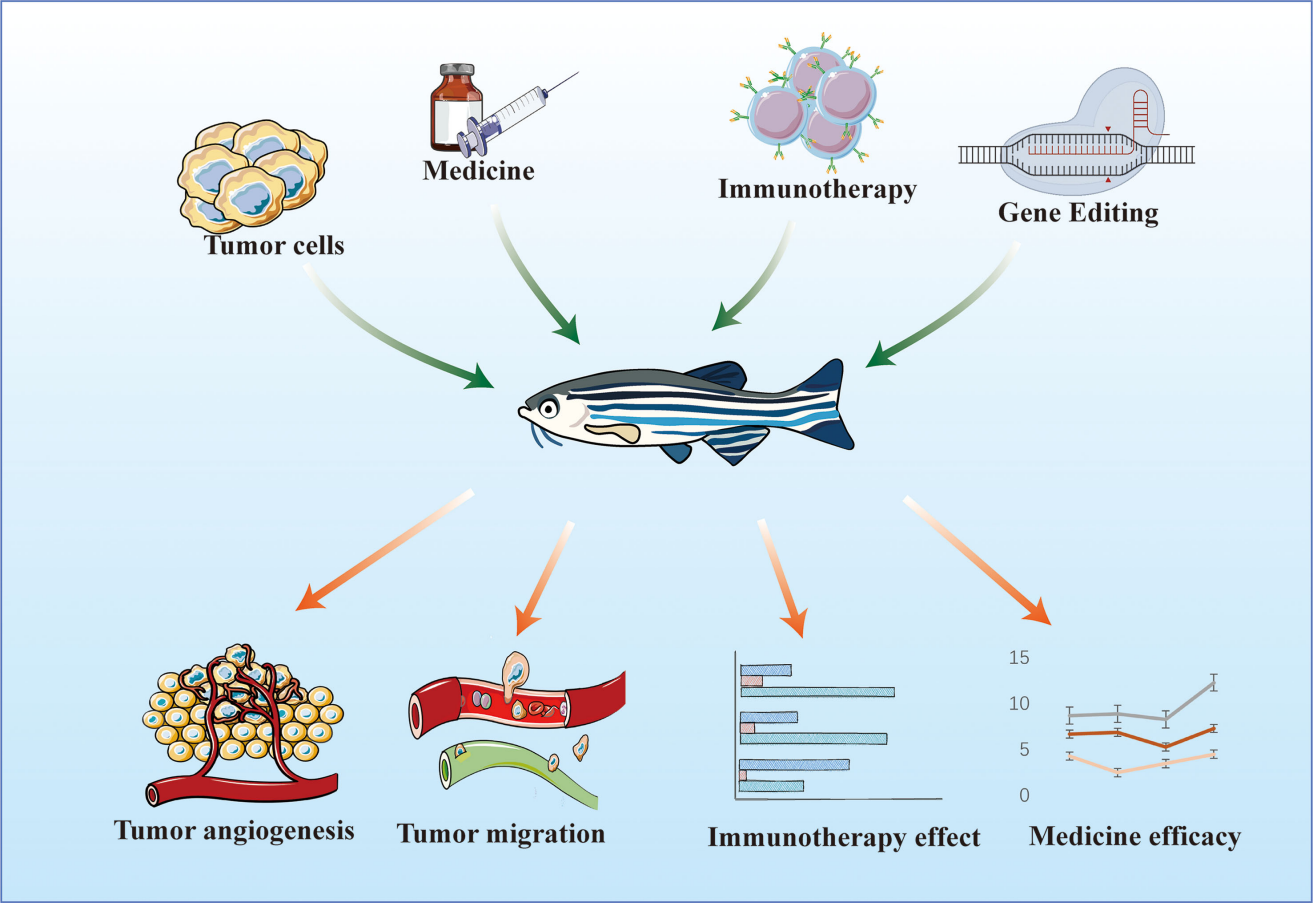
Zebrafish model for evaluating the effectiveness of antitumor therapy.

The research and development of novel drugs are long and costly processes. Drugs are first developed through *in vitro* tests, in which the effects of small-molecule therapies on cells are determined by detecting cell proliferation, cytotoxicity, marker expression, migration, signaling pathway activation, and morphological changes. Then, *in vivo* tests are conducted, in which the half-life of a drug is evaluated, and final drug screening is performed. Zebrafish are aquatic organisms that can absorb small-molecule compounds directly from water (63), and are thus more suitable for small-molecule drug screening and drug delivery pathway research than mice. In addition, a zebrafish has a short maturation period and produces a large number of offspring, and the cost of manipulating its genetic material is low. Thus, constructing tumor models from zebrafish is less expensive and time consuming than constructing tumor models from nude mice. Owing to the merits of zebrafish, it can be used as an intermediate evaluation model at the cellular and mammalian model levels for the rapid screening of antitumor active drug molecules and evaluation of the efficacy of antitumor therapies. The use of the zebrafish system spans from the discovery phase of high-content and high-throughput screening to the preclinical screening phase (64). We predict that zebrafish models will be increasingly used for drug assessment evaluation in the therapeutic context (**Figure 2**).

The use of zebrafish in drug discovery not only can improve the success rate of late-stage clinical drug development but also can reduce the cost and time required for screening (65). Owing to the high genetic affinity between zebrafish and humans, low breeding cost, convenient model construction, and easy genetic manipulation, which allows for the simultaneous construction of a large number of xenograft models for high-throughput drug screening, zebrafish is more suitable for high-throughput drug screening than other animal models. The short developmental process and fast metabolic efficiency of zebrafish can greatly reduce the time and increase the rate of drug screening. Thus, zebrafish xenografts have been used for the *in vivo* screening of drugs for CRC treatment, including cetuximab and regorafenib, and are effective and rapid *in vivo* model for human tumor drug trials (66).

## 4 Application of zebrafish modeling in the study of lncRNAs

### 4.1 Role and mechanisms of lncRNAs in tumor biology

Measurement and analysis of human transcriptional profiles have shown that the human genome is universally transcribed, but only 2% of RNAs encode proteins (67, 68). Transcripts greater than 200 nt in length have been widely categorized as lncRNAs (69–71). LncRNAs play crucial roles in cellular processes, including cell cycle regulation (72), cell differentiation and development (73), metabolism (74), immunity (75), and cancer genesis and development (76) and are potentially involved in viral infections (75, 77).

LncRNAs have various mechanisms of action, can regulate transcriptional and epigenetic modifications by interacting with DNA (78–80), participate in the regulation of translational and post-translational modifications through interactions with proteins (81, 82) and RNAs (83, 84), and can directly interact with signaling receptors (85). Although lncRNAs were previously categorized as noncoding RNAs, multiple lncRNAs contain open reading frames that produce micropeptides involved in a variety of biological processes and associated with many pathophysiological processes, such as carcinogenesis and tumor invasion and metastasis (86, 87) and innate immune responses (88). The discovery of lncRNA-derived peptides has generated a new mode of action by which these peptides interact with proteins and influence biological processes involved in the onset and progression of a disease, particularly cancer and immune diseases (89).

For example, large amounts of lncRNAs promote tumor growth and metastasis.PVT1 is required for high levels of c-Myc protein; PVT1 RNA and c-Myc protein expression are highly correlated in primary human tumors. Inhibition of PVT1 suppresses c-Myc-driven tumorigenic potency (90). MALAT1 was first identified in the lung cancer MALAT1 and is one of the most studied lncRNAs in cancer. For example, MALAT1 can promote tumorigenesis through the Wnt/β-catenin pathway, EMT, PI3K/AKT pathway, ERK/MAPK pathway and angiogenesis (91). HOTAIR alters histone (H3K27) methylation patterns and gene expression, thereby promoting tumor cell invasion. On the other hand, inhibition of HOTAIR decreases cell invasion. This is mainly attributed to the ability of HOTAIR to act as a scaffold for specific histone-modifying enzymes, affecting the expression of specific genes (92). Finally, LINK-A expression and activation of the LINK-Adependent signaling pathway are associated with triple-negative breast cancer (TNBC), and together they promote glycolytic reprogramming and tumorigenesis in breast cancer (93).

A number of lncRNAs have been identified in zebrafish (94, 95) and mice (96, 97); however, their primary sequences are weakly conserved between the species (98, 99). For example, few lncRNAs are conserved in the primary sequence between zebrafish and humans, and the phenotype of functionally inactivated zebrafish with conserved lncRNAs can be recovered using homologous genes from mouse or human (94). These results suggest that the higher-order structures of lncRNAs may be conserved, rather than their primary sequences. Thus, zebrafish may be a suitable model for exploring lncRNAs with function distinct from those indicated by the primary sequences.

### 4.2 Role of LncRNAs in the regulation of zebrafish tumor models

Since the development of zebrafish as a promising experimental model for human cancer research (100), many researchers have applied zebrafish model to determine the oncogenic role of lncRNAs in tumors. In the study of certain lncRNAs, zebrafish can be easily microinjected with Morpholinos at the embryonic and single-cell stage because zebrafish has homologous lncRNAs (101). Efficient screening of knockout or knockdown lines is ensured by the high number of fast-developing zebrafish zygotes, which facilitates investigations on the function of relevant lncRNAs throughout the course of tumor development and functional roles of relevant lncRNAs during normal development. Similar inferences have been drawn from cultured human and mouse cells (98). For example, in a study of the oncogenic role of the lncRNA THOR in melanoma, Hosono et al. (102), identified an exonic homolog of THOR in the zebrafish genome through bioinformatics prediction and later verified that zebrafish with knocked out THOR gene are resistant to melanoma formation, demonstrating that zebrafish can be used as a model for studying tumor-associated lncRNAs.

LncRNAs that undergo changes in expression in tumor tissues can be explored using a tumor xenograft model of zebrafish. One method is to construct a tumor xenograft model of zebrafish and then subject the zebrafish to different treatments, collect and analyze changes in the expression target lncRNAs, and ultimately validate the function of the relevant lncRNAs in tumor development. For example, in the study of STEAP3-AS1, a zebrafish *in situ* colorectal cancer model was constructed. Under normal or 8% oxygen condition, STEAP3-AS1 up-regulation may be a key factor in tumor development. AS1 upregulation may be required for hypoxia-mediated tumor metastasis (103). Another way is to treat cells used in tumor xenograft implantation models, such as knocking down, knocking out, and overexpressing relevant genes in tumor cells, before models are established for observation and data related to tumor proliferation and metastasis are collected; this approach allows for the validation of the *in vivo* functional roles of relevant lncRNAs. For example, Shen et al. (104) constructed a zebrafish xenograft model using lung cancer cells with knocked down PVT1, evaluated cell proliferation by quantifying the yolk fluorescence region of zebrafish larvae, and explored the migration of lung cancer cells with knocked down PVT1 by quantifying the trunk fluorescence region of zebrafish larvae; they verified that the growth and metastasis of lung cancer cells were inhibited when Lnc PVT1 was knocked down.

Furthermore, in the study of LncRNA LETS1, a zebrafish xenograft model was applied to verify that LETS1 deletion attenuated TGF-β-induced EMT and migration of breast and lung cancer cells *in vitro (* 105). Similarly, a zebrafish xenograft model was used to study the functions associated with the lncRNA SNHG4 in studies of colorectal cancer proliferation and migration (14). Although the results of studies applying the zebrafish xenograft model to study the functions associated with LncRNAs in different tumors are not yet abundant, a large number of relevant experiments are being carried out due to the advantages of the zebrafish xenograft model in relevant studies.

**Figure 3:**
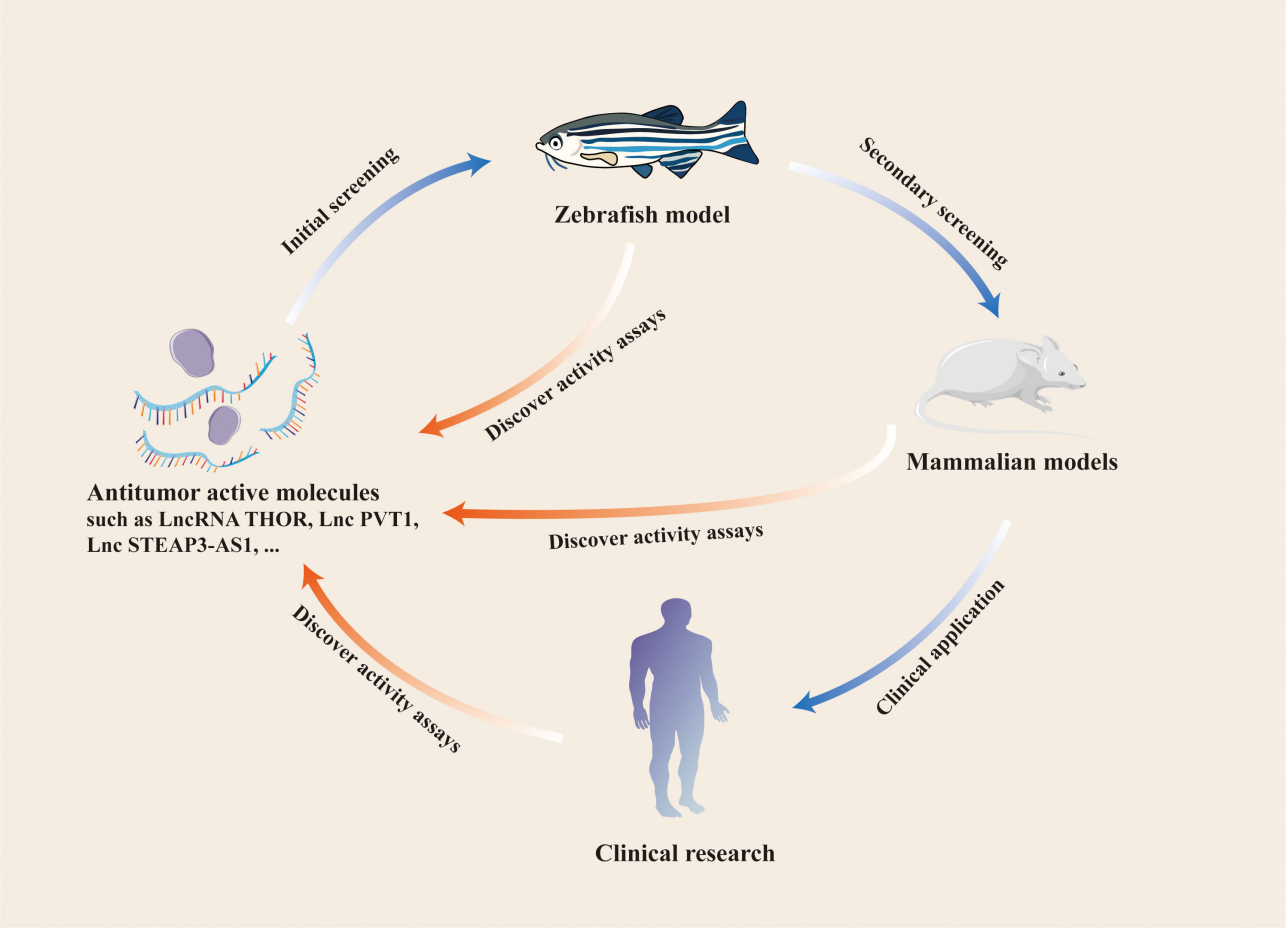
Application of zebrafish model in the study of LncRNAs and other antitumor active molecules.

The progression of tumor cells can be assessed with a zebrafish xenograft model, and the *in vivo* function of relevant lncRNAs can be verified 96 h after the model is constructed. By contrast, mouse xenograft models require 3–5 weeks. For studies on endogenous lncRNAs in tumors, shRNA plasmids for gene silencing must be constructed in a mouse model. The zebrafish model only requires the design and synthesis of specific siRNAs. When lncRNAs are examine as indicators of early tumor surveillance, the behavior of early tumor cells can be monitored *in vivo* according to the characteristics of the immature adaptive immune system of zebrafish larvae and the transparency of the body surface. These procedures are difficult to apply to mouse models. These results suggest that the zebrafish xenograft model is suitable for studying lncRNAs and monitoring tumor progression (**Figure 3**).

## 5 Conclusion

We summarize the development of zebrafish as a model organism, introduce the various growth and development stages of zebrafish, and illustrate the benefits of constructing tumor xenograft models of zebrafish. First, zebrafish tumor xenotransplantation facilitates research on tumor biology, and the proliferation and migration of tumor cells can be evaluated in a relatively short period of time after transplantation; relevant assays can be performed 3 days after drug treatment (49). Second, tumor growth and migration can be continuously monitored in zebrafish larvae in contrast to mouse models, allowing for the real-time observation of tumor cells’ behavior (106). In addition, transparent zebrafish embryos are essential, and single-cell resolution in zebrafish Casper lines exceeds that in mouse transplantation models, making prolonged observation feasible because fish do not need to be sacrificed for analysis (35, 56). These advantages support the use of zebrafish xenograft models in tumor research.

We also summarize and analyze some specific zebrafish xenograft models and their applications to studies on tumor angiogenesis and immunotherapy and drug screening (64, 107, 108). More importantly, we incorporate the application of lncRNAs in zebrafish model studies. Zebrafish xenografts can be used as *in vivo* tools for studying the role of lncRNAs in tumor cell proliferation and migration *in vivo* and preliminarily validating the relevant functions of lncRNAs in zebrafish models. Molecular mechanisms by which specific lncRNAs regulate the growth of tumors *in vivo* can be explored using these models, and some antitumor growth functional lncRNAs can be screened out and validated on mammalian models and in clinical trials (14, 102, 109). The zebrafish model has its own unique advantages over the mouse model, such as simple operation, high efficiency, and low cost. We compare the mouse model, the zebrafish model in more detail (**Table 2**). These advantages can facilitate the cellular level detection of antitumor active molecules. The *in vivo* environment of a zebrafish model can mimic the growth characteristics of human tumors and ensures the accurate and efficient identification of effective antitumor active molecules.

**Table 2:** Comparison of zebrafish model with mouse model.

Model type	Cost	Experimental period	Number of cells required per individual	Drug Screening Flux	Formation of solid tumors
Zebrafish model in larvae	low	5 – 7 days	10^2^	high	no
Zebrafish model in adults	low	weeks to months	10^5^	medium	no
Mouse model	high	weeks to months	10^5^ - 10^6^	low	yes

Despite the many advantages of the zebrafish tumor xenograft model, there are still some shortcomings. First, it is obvious that zebrafish are devoid of lung, mammary, and prostate organs, and it is not possible for us to directly study primary cancers in these organs on zebrafish models (although relevant xenograft models can be constructed). Second, most transgenic zebrafish models are still based on transgenic expression of human oncogenes rather than knock-in of endogenous or homologous zebrafish loci, and thus may not have physiologic expression levels. Finally, in transplantation models of zebrafish larvae, the small size limits the number of transplanted cells to 100-200 per fish, which often fails to encompass cancer-driving stem cells and accurately recapitulates the genetic heterogeneity and drug-responsive behavior driven by rare subclones in human tumors.

Zebrafish models cannot completely replace mammalian models, such as mice, but can be used as a bridge between the cellular-level and mammalian models *in vivo*, thus greatly contributing to the progress of related studies and reducing research cost. Overall, the zebrafish xenotransplantation model is a reliable and promising model for human cancer research, and transplantation models are reliable and promising models for human cancer research.
